# How SARS-CoV-2 Big Data Are Challenging Viral Taxonomy Rules

**DOI:** 10.3390/v15030715

**Published:** 2023-03-09

**Authors:** Daniele Focosi, Fabrizio Maggi

**Affiliations:** 1 North-Western Tuscany Blood Bank, Pisa University Hospital, 56126 Pisa, Italy; 2 National Institute for Infectious Diseases “L. Spallanzani”-IRCCS, 00149 Rome, Italy

SARS-CoV-2 genomic sequencing has peaked to unprecedented compared to other viruses. In three years, GISAID has included more than 15 million SARS-CoV-2 sequences. Such big data are facilitating the construction of detailed phylogenies and will likely represent a model for coming epidemics and pandemics, but they are also posing incredible challenges.

There is currently much debate about the sustainability of phylogenetic nomenclature systems for SARS-CoV-2 variants. The PANGO phylogeny currently represents the most detailed system [1], and so far it has definitely helped tracking emerging variants [2], but lacks homogeneous criteria for designation, including a human factor in designation and thus being prone to biases and delays. In this regard, an automated agnostic designation based on growth rates has been proposed as a solution [3], as well as automated pipelines for the identification of recombinant lineages [4]. The growing number of discontinuous aliases for Omicron (accounting for 683 sublineages designated since 7 December 2021, to 23 January 2023) makes rooting difficult to remember, e.g., who can remember that XBB.1.9.2 corresponds to the recombinant between B.1.1.529.2.10.1.1 and B.1.1.529.2.75.3.1.1.1, and who can actually remember that EG.1 is a direct descendant of XBB.1.9.2? All these troubles raise the question: has PANGO designated too much in the last few months or is the scheme collapse-prone in the coming months?

On the other side, the WHO nomenclature for variants of concern (VOC) is prone to saturation (a few letters left available within the Greek alphabet), lacks transparent criteria for designation (are not the recently described Chinese variants driving the wave of concern?), and is stuck at Omicron since more than one year despite the many subsequent relevant waves that have occurred after BA.1 [5] (Figure 1). This steadiness has unfortunately created the basis for the minimizers’ statement that “it is all Omicron and Omicron is mild”.

The NextStrain naming system stems from the WHO VOC/VOI definition [6] but is definitely more transparent and objective (>20% global frequency for ≥2 months, >30% regional frequency for ≥2 months; >0.05 per day growth in frequency where it is circulating and has reached >5% regional frequency). Nevertheless, NextStrain still suffers from delays (frequencies occurring much after growth advantages are clear), arbitrary cutoffs, and the vague definition of “region” (again, is not China a region?).

Mythological names have been suggested on Twitter to facilitate reporting on media, but they are equally prone to saturation and memory pain, and can eventually cause unjustified panic and inurement.

The search for alternative and simple phylogenies is therefore still ongoing and could benefit from phenotype parameters. A definition of serotypes based on antigenic distance using convalescent sera is a possible approach: antigenic cartography clearly shows that XBB.* and BQ.1.* differ from D614G SARS-CoV as much as from SARS-CoV, and would hence deserve a separated name [7,8]. In any case, with repeated exposures to both infection and vaccination and the consequential dynamic hybrid immunity, the implications of such a naming scheme for viral ecology and public health are likely to be puzzling. Stratification by virulence in unvaccinated animal models is another possible approach, but so far each animal model (either humanized or natural) shows substantial differences with pathology in humans [9].

The real question is if and when SARS-CoV-3 could be declared. According to the International Committee for Taxonomy of Viruses (ICTV), SARS-CoV-2 is not a species per se but rather a member of the species “SARS-related coronavirus” under the subgenus “Sarbecovirus”. Such species include not only SARS-CoV-2 but also SARS-CoV and many more bat or civet coronaviruses [10]. Then, SARS-CoV-2, and eventually SARS-CoV-3, could be more easily defined as subspecies.

Some researchers previously advocated that Omicron (BA.1) was already so different from Delta that it was not worth the SARS-CoV-3 designation. While the concept of species sounds out of scope for viruses not having sexual reproduction, the current (2013) definition of species issued by the International Committee for Taxonomy of Viruses (ICTV), included within the International Code of Virus Classification and Nomenclature, varies according to genus and is based on multiple criteria. For most genera, a mixture of evolutionary and non-evolutionary variables (such as geographic distribution, host range, or symptomatology) are considered to define a species [11]. Genetic distance, as applied by NextStrain for SARS-CoV-2 and many other viruses (https://nextstrain.org/pathogens, accessed on 14 September 2022), is apparently a more reliable parameter, despite currently varying across genera and based on arbitrary cutoffs (e.g., 30% for Ebolaviruses, 89% for Gemyniviruses).

The absence of the SARS-CoV-3 name is most likely due to a desire to avoid mass panic, a criterion that is anything but evolutionary. Each phylogenetic scheme clearly has its own advantages and disadvantages, and finding a solution will be difficult. For example, even a minor genetic distance can have a large impact on immune escape and thus public health, as demonstrated by influenza viruses [12], and most of the differences between SARS-CoV-2 VOCs have been restricted to a few amino acids within the receptor-binding domain. However, we need to move on. Hiding our heads in the sand will not protect us from the ongoing viral evolution.

## Figures and Tables

**Figure 1:**
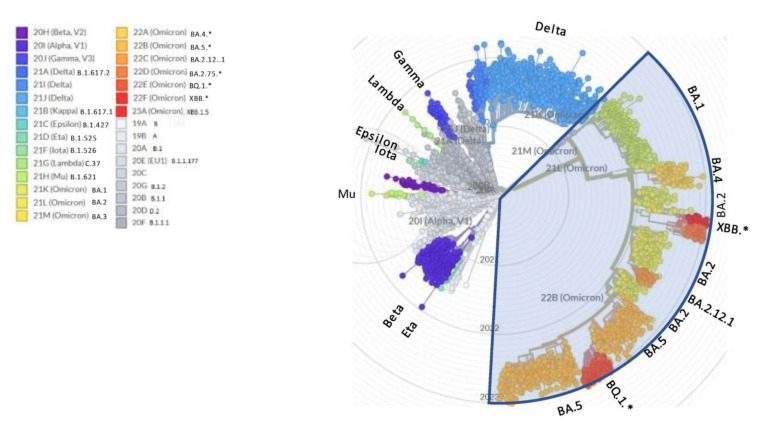
NextStrain radial view of SARS-CoV-2 phylogenetic tree. The legend shows the corresponding WHO variants of concern (VOC) and PANGOLIN names. * asterisk indicates that sublineages are included in the variant.
